# Influence of seating styles on head and pelvic vertical movement symmetry in horses ridden at trot

**DOI:** 10.1371/journal.pone.0195341

**Published:** 2018-04-05

**Authors:** Emma Persson-Sjodin, Elin Hernlund, Thilo Pfau, Pia Haubro Andersen, Marie Rhodin

**Affiliations:** 1 Department of Anatomy, Physiology and Biochemistry, Swedish University of Agricultural Sciences, Uppsala, Sweden; 2 Department of Clinical Science and Services, The Royal Veterinary College, North Mymms, Hatfield, United Kingdom; 3 Department of Clinical Sciences, Swedish University of Agricultural Sciences, Uppsala, Sweden; University of Illinois, UNITED STATES

## Abstract

Detailed knowledge of how a rider’s seating style and riding on a circle influences the movement symmetry of the horse’s head and pelvis may aid rider and trainer in an early recognition of low grade lameness. Such knowledge is also important during both subjective and objective lameness evaluations in the ridden horse in a clinical setting. In this study, inertial sensors were used to assess how different rider seating styles may influence head and pelvic movement symmetry in horses trotting in a straight line and on the circle in both directions. A total of 26 horses were subjected to 15 different conditions at trot: three unridden conditions and 12 ridden conditions where the rider performed three different seating styles (rising trot, sitting trot and two point seat). Rising trot induced systematic changes in movement symmetry of the horses. The most prominent effect was decreased pelvic rise that occurred as the rider was actively rising up in the stirrups, thus creating a downward momentum counteracting the horses push off. This mimics a push off lameness in the hindlimb that is in stance when the rider sits down in the saddle during the rising trot. On the circle, the asymmetries induced by rising trot on the correct diagonal counteracted the circle induced asymmetries, rendering the horse more symmetrical. This finding offers an explanation to the equestrian tradition of rising on the ‘correct diagonal.’ In horses with small pre-existing movement asymmetries, the asymmetry induced by rising trot, as well as the circular track, attenuated or reduced the horse’s baseline asymmetry, depending on the sitting diagonal and direction on the circle. A push off hindlimb lameness would be expected to increase when the rider sits during the lame hindlimb stance whereas an impact hindlimb lameness would be expected to decrease. These findings suggest that the rising trot may be useful for identifying the type of lameness during subjective lameness assessment of hindlimb lameness. This theory needs to be studied further in clinically lame horses.

## Introduction

Disorders of the locomotor apparatus are common in the riding horse population [1]. Retrospective studies of pre-purchase examinations showed that 20–53% of the horses were found lame [2–4]. It can be assumed that the majority of sellers and buyers were unaware of such lameness. Greve and Dyson [5] subjectively evaluated 506 horses considered sound by their owners and found that 46% of the horses were lame. Objective symmetry measurements using inertial sensors found that 73% of 222 riding horses—perceived as free from lameness by their owners—had movement asymmetries, of which many were in the same range as measured in horses deemed to be lame [6]. This high prevalence of asymmetries may indicate that many horses are ridden despite the presence of orthopaedic pathology, with a subsequent increased risk of developing chronic orthopaedic disease.

One possible reason for the apparent lack of recognition of lameness may be that the rider’s seating style influences the movement asymmetry of the horse. Such rider induced asymmetries may complicate the perception of lameness for both rider and observer.

Due to its important welfare implication many aspects of horse-rider interaction has been studied [7] but to a lesser extent the rider’s influence on vertical movement symmetry of the horse. The dorsoventral movement of the spine will be decreased with the presence of a rider in both sitting and rising trot [8]. In rising trot, also known as posting, the rider descends from the standing position to sit down in the saddle during the second part of the stance phase of one diagonal pair of limbs, to subsequently rise up during the end of that same stance to stand up in the stirrups during the stance phase of the other diagonal. This uneven movement of the rider produces an uneven biphasic load that affects the motion symmetry of the horse’s pelvis and lumbar back [9]. During rising trot on circles or turns, the equestrian society defines the correct diagonal as the rider sitting down when the outside forelimb and inside hindlimb are in stance (i.e. on a right circle sitting down during left forelimb-right hindlimb stance and standing up in the stirrups during right forelimb–left hindlimb stance). The reason for this definition is not described.

In rising trot on a circle, on the correct diagonal, mainly pelvic asymmetries but also head movement asymmetries, although to a lesser extent, have been observed [10]. In addition, lungeing on a circle will induce systematic changes in movement symmetry of head and pelvis in both sound and lame horses [11–13]. In horses with pre-existing lameness (asymmetry score above 0.05) Licka et al. [14] showed that the presence of a rider in sitting trot on a straight line did not alter the degree of forelimb lameness. The degree of hindlimb lameness did increase under an experienced rider but the type of lameness (impact or push off) was not described [14].

To our knowledge, the effect of rider seating style on pre-existing motion asymmetries while being ridden in rising trot and on a circle has not yet been studied. We believe that an increased knowledge of how the rider seating style as well as riding on a circle influences movement symmetry of the horse may aid observers and riders in detection of low grade lameness during training. This is also important knowledge for subjective or objective lameness evaluations of horses being ridden.

Therefore, the aim of this study was to objectively quantify how the rider’s seating style (sitting, two point seat or rising trot) influences the movement symmetry in trotting horses, in a straight line and on a circle. Our hypothesis was that rising trot and riding on a circle induce systematic changes in the movement symmetry of the horse. We also tested a post-hoc hypothesis that these asymmetries would attenuate or reduce any pre-existing movement asymmetries depending on whether the seating style- or circle-induced asymmetries agree or disagree in direction with the pre-existing asymmetry.

## Materials and methods

In this study, inertial sensors were used to assess the effect of rider seating style on vertical movement symmetry in 26 riding horses trotting on the straight and on the circle in both directions, totaling three movement directions. The study protocol was approved by the Ethical Committee for Animal Experiments, Uppsala, Sweden and informed consent was obtained from the Swedish National Equestrian Centre, Strömsholm, who owned all the horses.

Horses included (n = 26) were 18 geldings and 8 mares of Swedish Warmblood breed, training at intermediate level in dressage and/or showjumping; mean (range) age 12 years (8–18 years), height at withers 168 cm (161–175 cm), body mass 600 kg (540–658 kg). The horses had not been treated for lameness for the six months preceding the data collection and were considered free from lameness according to their owner. During the experiment all horses were ridden by the same, left-handed, intermediate level dressage rider (height 178 cm and body mass 60 kg), and wore their usual saddle and bridle with a snaffle bit.

### Study protocol and data collection

The horses were subjected to 15 different conditions, trotting on the straight and on the circle in both directions, totaling three movement directions. There were three unridden conditions and 12 ridden conditions where the rider performed three different seating styles (the rising trot style was performed on both diagonals, producing four seating conditions). First, ‘sitting:’ sitting trot with short reins (contact with the horse “on the bit”). Second, ‘two point seat:’ light seat with loose reins (no contact). Third, ‘rising trot:’ with short reins where the rider descends from the standing position to sit down in the saddle during the second part of the stance phase of one of the diagonal limb pairs. The rising trot was performed both with the rider descending during the right forelimb (RF)- left hindlimb (LH) diagonal stance and during the left forelimb (LF)- right hindlimb (RH) diagonal stance (henceforth simply referred to as ‘sitting during RF/LH diagonal stance’ or ‘sitting during LF/RH diagonal stance’). On a circle, the equestrian society defines the correct diagonal for rising trot as sitting down when the outside forelimb and inside hindlimb are in stance (i.e. on a right circle sitting during left forelimb-right hindlimb stance and rising during left forelimb–right hindlimb stance). These seating styles were then combined with the movement directions to create the following 15 conditions (see Table 1).

**Table 1:** List of conditions and combined conditions for first mixed model.

Condition	Combined condition
**Unridden trials**	**Unridden trials**
‘unridden straight'	unridden straight
‘unridden circle right'	unridden circle
‘unridden circle left'	unridden circle
**Ridden trials**	**Ridden trials**
‘ridden straight, sitting'	straight line sitting
‘ridden straight, two point seat	straight line two point seat
‘ridden straight, rising trot, rider sitting during RF/LH diagonal stance'	straight line rising trot
‘ridden straight, rising trot, rider sitting during LF/RH diagonal stance'	straight line rising trot
‘ridden circle right, sitting'	circle sitting
‘ridden circle right, two point seat'	circle two point seat
‘ridden circle right, rising trot, sitting on correct diagonal (LF/RH)'	circle rising trot correct diagonal
‘ridden circle right, rising trot, sitting on incorrect diagonal (RF/LH)'	circle rising trot incorrect diagonal
‘ridden circle left, sitting'	circle sitting
‘ridden circle left, two point seat’	circle two point seat
‘ridden circle left, rising trot, sitting on correct diagonal (RF/LH)'	circle rising trot correct diagonal
‘ridden circle left, rising trot, sitting on incorrect diagonal (LF/RH)'	circle rising trot incorrect diagonal

The definitions of the 15 conditions performed and the 9 combinations of these (combined conditions) used in the first mixed model analysis.

The horses were randomly allocated to start with the unridden or ridden conditions and the order of sub-categories of these were randomly assigned to each horse. Both sets of conditions started with 5 min of warm-up (ridden or lunged). Whenever the horses were tripping, changing gait or pulled excessively on the lunge line/lead rope, the condition was repeated until a data collection, free from such unwanted behavior, was completed.

### Instrumentation

The horses were fitted with a commercially available inertial measurement unit (IMU) system (Lameness Locator, Equinosis TM) consisting of three sensors. One single axis (vertical) accelerometer with a range of 6 g was attached to the poll with a felt head bumper attached to the bridle. Another sensor was taped to the midline of the pelvis between the two tubera sacrale. The last and third sensor, a single axis (sagittal plane) gyroscope sensor with a range of 300°/s, was secured to the dorsum of the right forelimb pastern using a specially designed pastern wrap. Each sensor measured 3.4 x 3.1 x 2.0 cm and had a mass of 31g. Data were digitally recorded (8bits) at 200Hz and wirelessly transmitted to a handheld computer.

Movement symmetry was measured with the IMU system described above in the same indoor arena (sand and fibre surface). The straight line measurements were performed back and forth approximately 60m in length, each way, along the long side of the arena. The circle conditions were performed on a 12m diameter circle (size controlled by marker placed on the lunge line or by markings on the ground).

### Data processing

The sensor data were analyzed with the software included in the IMU system. The recorded vertical acceleration was converted to vertical displacement using a moving-window, error correcting, double integration algorithm as described by Keegan et al. 2001 and 2011 [15, 16]. Angular velocity data from the gyroscope was used for stride splitting. The two local displacement minima and maxima for the head and pelvis in each stride were identified. Trial means of the stride-to-stride difference of the minimum head (HDmin) or pelvic (PDmin) height between right and left limb stances were calculated. Similarly HDmax and PDmax were calculated as the trial means of the stride-by-stride difference of the maximum head or pelvic height prior to right limb stance minus the maximum head or pelvic height prior to left limb stance [15]. Positive values thus indicate less downward movement during stance or less upward movement produced in the push off phase (lower maximum position reached) of the right forelimb or hindlimb. Thus positive values represent asymmetries attributed to less load absorption/force production of the right forelimb/hindlimb, and negative values represent asymmetries attributed to ditto of the left forelimb/hindlimb. Removal of single outliers (up to maximum 10% of the strides) for the head movement was performed in the software package.

### Statistical analysis

Data from all horses were analyzed using SAS statistical software® (SAS Institute). Mixed models were created using the procedure Proc Mixed with symmetry variables HDmin, HDmax, PDmin and PDmax (trial means) as outcome. All ridden and unridden circle conditions were combined by mirroring the measurements from left circle conditions (they were multiplied by -1). This allowed analysis of the main effect of the horses’ movements on a circle independent of their individual differences between directions and the main effect of the rider sitting on the correct and incorrect diagonals in relation to circle direction. Rising trot on both diagonals in a straight line were also combined by mirroring measurements where the rider was sitting during LF/RH diagonal stance. This resulted in a reduction (from originally 15) to 9 conditions evaluated in the first mixed model. Due to this reduction of conditions in the statistical analysis the direction of the asymmetry (positive vs negative) in the outcome represents the asymmetries presented during straight line rising trot with the rider sitting down during RF/LH diagonal stance and during ridden and unridden circles in the right direction. The variable ‘combined condition’ (see Table 1) was entered as a fixed effect and the repeated evaluation of each horse and the movement direction (left/right/straight) within each horse was represented by a random intercept effect in each model. Each symmetry variable was investigated for a reasonable transformation close to normality (i.e. transforming along the ladder of powers, [denoting the response variable as x: -1/x^2^, -1/x, -1/square-root(x), log (x), square-root(x), x and x^2^]) and plots of residuals scrutinized for normality. Evaluation of statistical significance was made using type III p-values. Pairwise comparisons (option pdiff in SAS) were performed for all conditions against the unridden straight condition, and also the ridden circle conditions were compared to the unridden circle. The level of significance was set as P<0.05.

Lame horses were not intentionally sought out in this study. However, in order test the post-hoc hypothesis, a second set of mixed models were created for a subset of horses selected for pre-existing asymmetry. Horses were deemed to show asymmetrical movement if they showed values exceeding the recommended thresholds for repeatable asymmetries for the head (absolute value ≥ 6 mm) or pelvic (absolute value ≥ 3mm) variables [15] and were thus included in the mixed model of that variable. One mixed model was created for each of the symmetry variables HDmin, HDmax, PDmin and PDmax. To enable a combination of left- and right sided asymmetric horses in the same analysis all values for the asymmetry variable (outcome variable) were multiplied by -1 for the horses with negative asymmetry values during ‘unridden straight’, effectively switching the sign of the asymmetry. This artificially rendered all baseline asymmetries positive (i.e. attributed to the RF or RH). Horse was entered as a random effect. The fixed effect was condition re-labeled to describe the position of the limb to which the asymmetry was attributed, in relation to the direction and the rider’s seat (see S1 File). Normality was checked as described above and pairwise comparisons were made with ‘unridden straight’ using the same principles as above.

## Results

From the 26 horses, 390 trials were collected, three trials in total were excluded; for one horse, two trials (‘ridden circle left, sitting’ and ‘ridden circle right, sitting’) were excluded due to lack of compliance with the desired condition (sitting trot with contact in reins not possible) and for another horse one trial (‘ridden straight, rising trot sitting during RF/LH diagonal stance’) was excluded due to unsuccessful data collection. During the baseline condition, ‘unridden straight', eighteen of the horses presented with one or more head and/or pelvic asymmetry variables above the previously mentioned thresholds. Mean ± standard deviation of HDmin, HDmax, PDmin and PDmax for all individual horses during ‘unridden straight’ are given in Table 2. In the statistical analysis, all symmetry variables were kept untransformed except for HDmin in the model using the subset of horses with asymmetrical movement where square transformation was deemed most appropriate.

**Table 2:** Baseline asymmetry.

Horse	Head		Pelvis		Horse	Head		Pelvis	
	HDmin	HDmax	PDmin	PDmax		HDmin	HDmax	PDmin	PDmax
**1**	-3.7	-5.7	-2.9	-1.3	**14**	-0.7	1.4	3.3*	-3.6*
**2**	1.5	8.3*	0.2	2.9	**15**	10.4*	-8.1*	-3.0	-3.9*
**3**	3.8	3.4	-0.6	-1.5	**16**	1.5	-2.1	1.6	4.7*
**4**	4.2	-1.0	-0.2	2.9	**17**	3.6	-3.6	1.0	-1.1
**5**	-2.6	6.2*	1.3	-3.6*	**18**	3.3	2.7	3.1*	-2.7
**6**	-5.0	-1.0	-0.5	1.4	**19**	-7.9*	-1.9	-5.9*	-1.2
**7**	10.3*	4.0	-3.0	-0.4	**20**	7.7*	0.0	3.3*	9.0*
**8**	-0.3	8.2*	-4.4*	4.5*	**21**	8.8*	9.3*	5.1*	1.1
**9**	-1.6	2.9	-2.2	-1.4	**22**	-2.6	2.0	0.2	-0.1
**10**	-1.4	-9.2*	4.1*	0.0	**23**	-0.5	-10.7*	1.9	-0.1
**11**	0.0	3.3	-1.5	0.5	**24**	2.1	2.5	3.7*	1.4
**12**	6.1*	-1.9	1.8	2.0	**25**	2.3	16.3*	-3.2*	-3.0*
**13**	2.8	5.6	-4.9*	-5.0*	**26**	0.6	-3.8	**-4.3**	1.4

* Boldface: Head or pelvic mean asymmetry variables with an absolute value of HDmin/HDmax of ≥ 6 mm or PDmin/PDmax of ≥ 3 mm.

Mean ± standard deviation of head and pelvic vertical asymmetry variables HDmin, HDmax, PDmin and PDmax for all individual horses during the baseline ‘unridden straight’ condition. Positive values indicate less downward movement during stance or less upward movement (lower maximum position reached) after the right (fore or hind) limb and negative values during/after the left (fore or hind) limb.

### Effects of conditions

**Fig 1:**
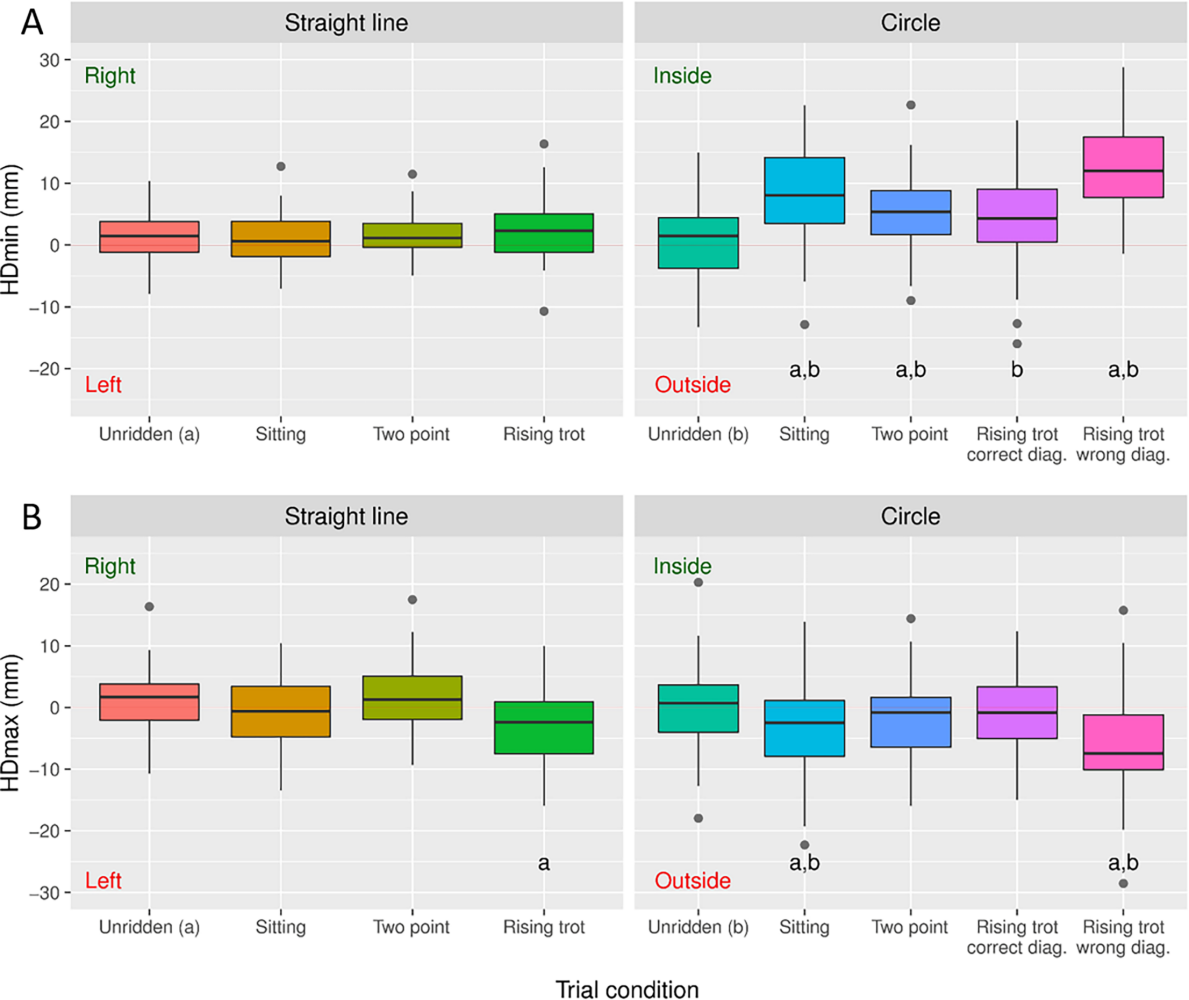
Head movement asymmetry per conditions. The mean difference in vertical minimum and maximum heights during/after right and left stance phases in trot for the head ((A) HDmin and (B) HDmax) are presented per condition. The gray dots indicate outliers. Significant differences from ‘unridden straight’ is indicated by **a**. Significant differences from’unridden circle’ is indicated by **b**. These pairwise comparisons were performed on mixed model output. **Right** and **Left** indicates right sided (positive) and left sided (negative) attributed asymmetries respectivly. **Inside** and **Outside** indicates the assymmetry being attributed to the inside (positive) and outside (negative) forelimb respectivly. Note that during rising trot in a straight line the rider is sitting down during the stance of RF/LH and that circles are performed to the right. This is due to the mirroring of data of the left circle and straight line rising trot on the opposite diagonal.

**Fig 2:**
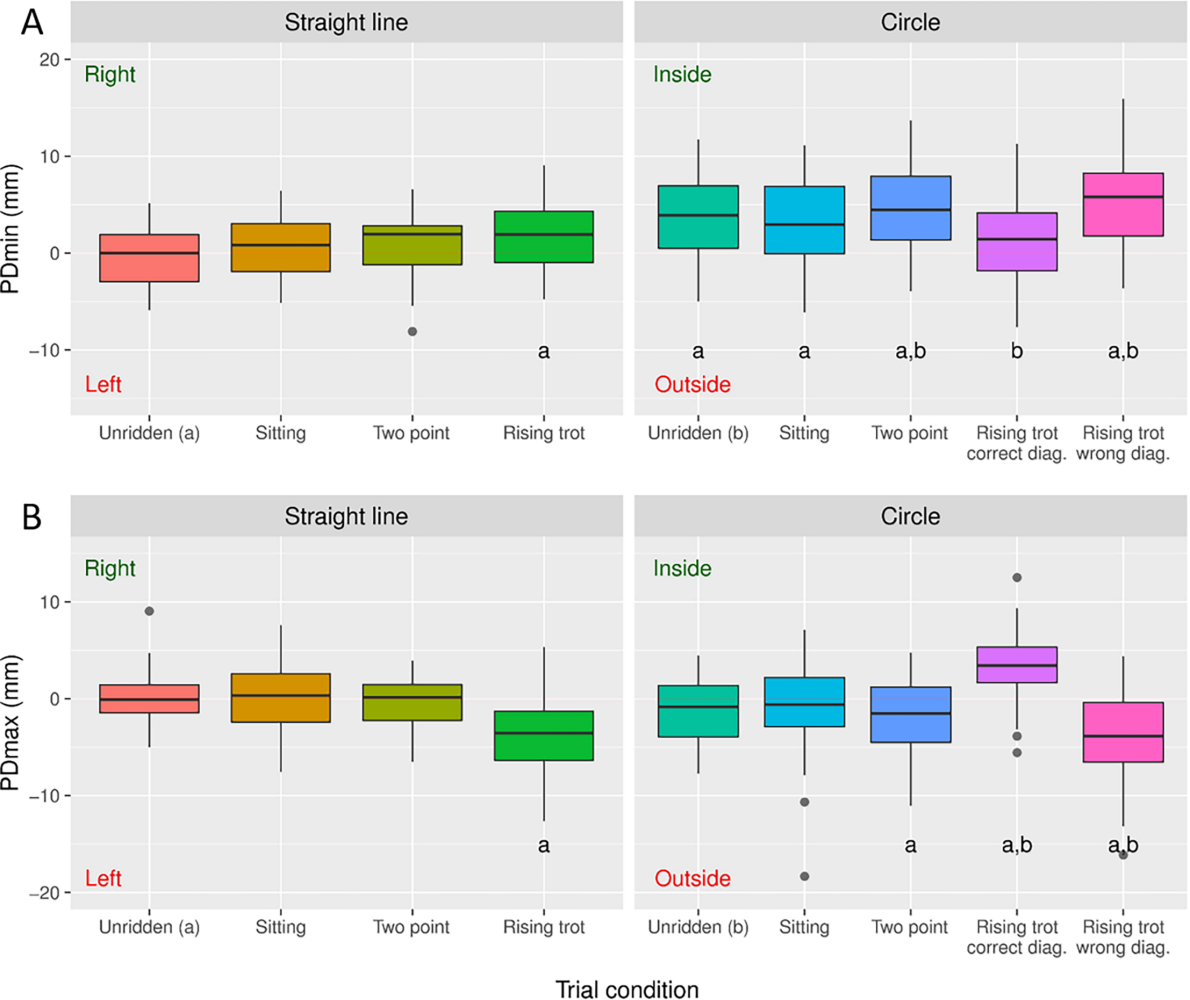
Pelvic movement asymmetry per conditions. The mean difference in vertical minimum and maximum heights during/after right and left stance phases in trot for the pelvis ((A) PDmin and (B) PDmax) are presented per condition. The gray dots indicate outliers. Significant differences from ‘unridden straight’ is indicated by **a**. Significant differences from’unridden circle’ is indicated by **b**. These pairwise comparisons were performed on mixed model output. **Right** and **Left** indicates right sided (positive) and left sided (negative) attributed asymmetries respectivly. **Inside** and **Outside** indicates the assymmetry being attributed to the inside (positive) and outside (negative) hindlimb respectivly. Note that during rising trot in a straight line the rider is sitting down during the stance of RF/LH and that circles are performed to the right. This is due to the mirroring of data of the left circle and straight line rising trot on the opposite diagonal.

The effect of the 15 different conditions (with the two circle directions and two rising diagonals combined in the statistical analysis resulting in 9 conditions presented) on head and pelvic symmetry variables across all horses are presented in Figs 1 and 2 and as mixed model output in the supplementary material (S2 File).

### Effect of conditions in horses with small pre-existing movement asymmetries

**Fig 3:**
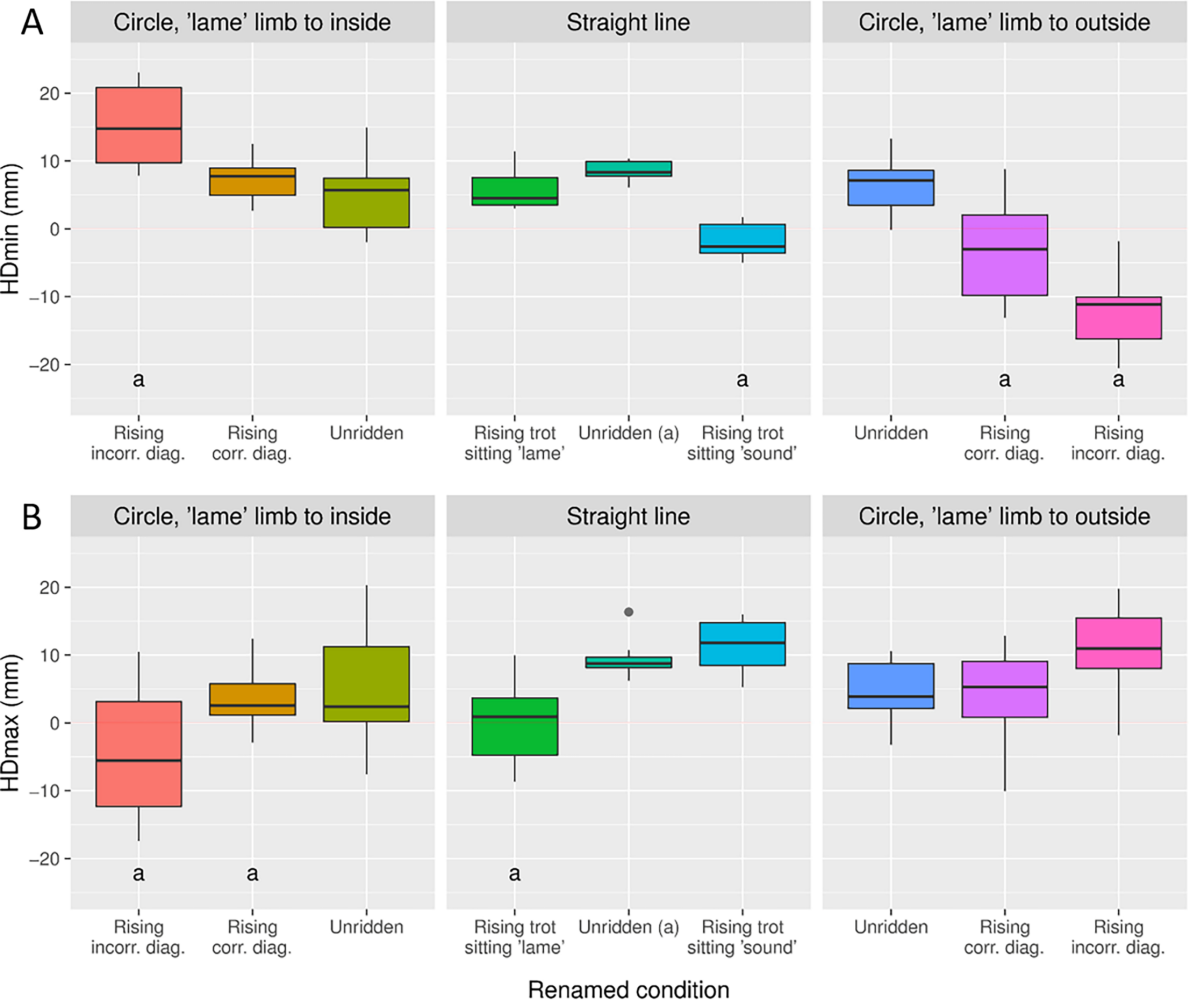
Head movement asymmetry per condition in the horse subsets selected for asymmetry. Each condition was labelled to describe the position of the forelimb that the asymmetry was attributed to in relation to the movement direction and the rider’s seating style. For example ‘lame’ limb to inside means that the forelimb the asymmetry is attributed to is on the inside of a circle. Rising trot sitting ‘lame’ means that the rider sits down during the stance phase of the ‘lame’ forelimb. Conditions with two point seat and sitting trot are not shown. Significant differences from unridden straight is indicated by **a.** Pairwise comparisons between trial conditions were performed on mixed model output. Since left and right asymmetries were combined in the statistical analysis, the direction (positve vs negative) of the asymmetries seen represents those for a horse with a right forelimb attributed asymmetry.

**Fig 4:**
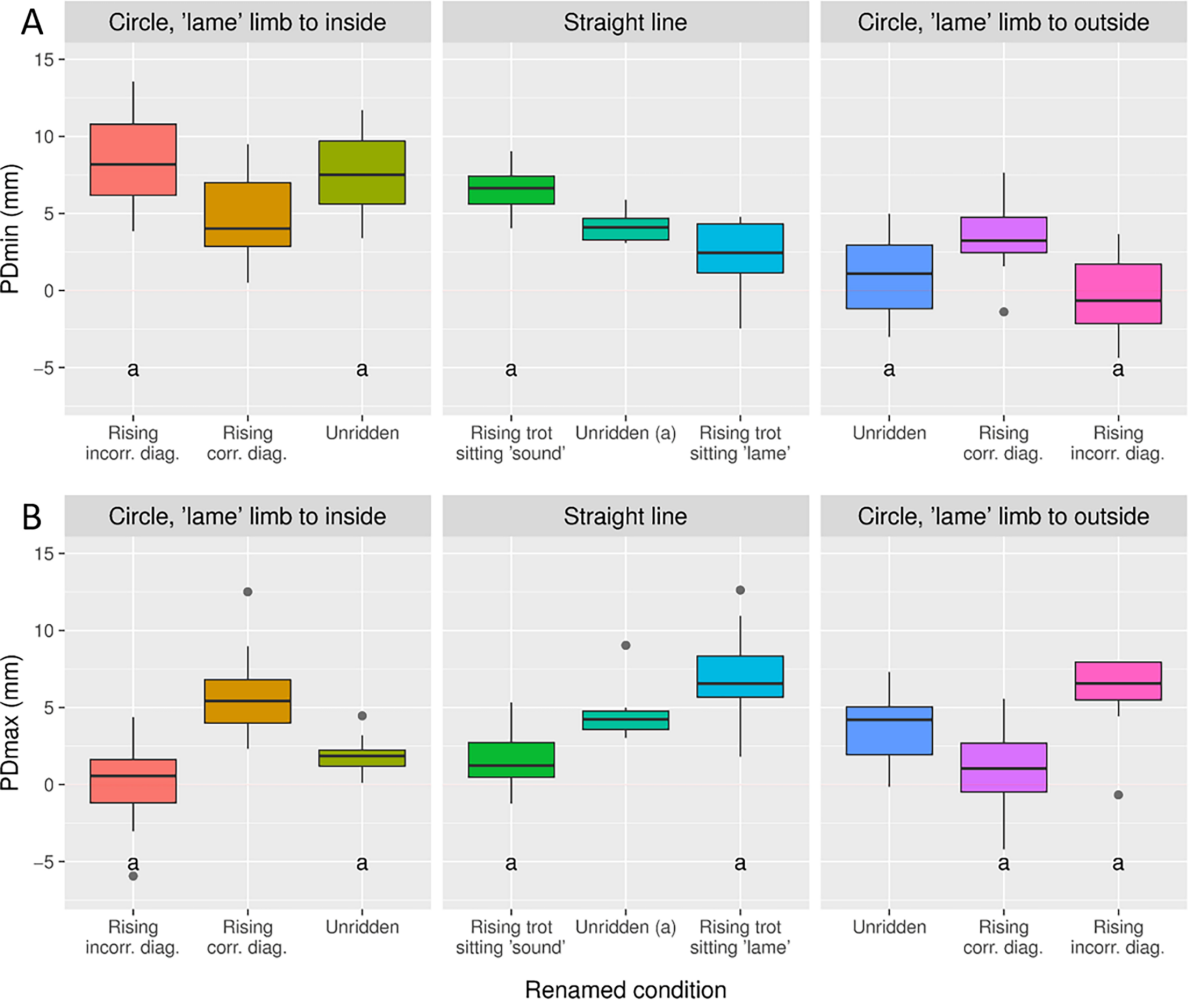
Pelvic movement asymmetry per condition in the horse subsets selected for asymmetry. Each condition was labelled to describe the position of the hindlimb that the asymmetry was attributed to in relation to the movement direction and the rider’s seating style. For example ‘lame’ limb to inside means that the hindlimb the asymmetry is attributed to is on the inside of a circle. Rising trot sitting ‘lame’ means that the rider sits down during the stance phase of the ‘lame’ hindlimb. Conditions with two point seat and sitting trot are not shown. Significant differences from unridden straight is indicated by **a.** Pairwise comparisons between trial conditions were performed on mixed model output. Since left and right asymmetries were combined in the statistical analysis, the direction (positve vs negative) of the asymmetries seen represents those for a horse with a right hindlimb attributed asymmetry.

Data for horses with small pre-existing movement asymmetries during baseline measurements are presented in Figs 3 and 4 and as mixed model output in the supplementary material (S3 File).

## Discussion

In the present study, we assessed the influence of commonly used rider seating styles in combination with straight and circular tracks on vertical movement symmetry in riding horses during trot. As a post-hoc hypothesis, we also investigated the effect on small pre-existing movement asymmetries in a subset of the horses.

### Effect of sitting trot and two point seat

Riding in straight line trot in a symmetrical seating style, such as sitting trot or two point seat did not influence the investigated vertical movement symmetry of the head and pelvis, neither in the complete dataset nor in the subset of horses with small pre-existing movement asymmetries. This is in agreement with Licka et al. [14], suggesting that the symmetrical load induced by the rider throughout the stride cycle in these positions does not affect the horse’s vertical movement symmetry. However in contrast to the present study, Licka et al. [14] did demonstrate an increased asymmetry in the hindlimb lame horses when ridden by an experienced rider, not seen under a novice rider. We do not know whether the differences in the results between these two studies are related to the origin and degree of hindlimb asymmetries, or an effect of the riders and their demands on the horse, such as degree of collection or static or dynamic rider asymmetry.

### Effect of rising trot

In contrast to symmetrical seating styles the rider movements during rising trot was shown to significantly influence the movement symmetry of the horse. The pelvis reached a lower minimum position during the stance phase when the rider was sitting, likely reflecting an increased loading of that hindlimb (Fig 2A). This is in accordance with Roepstorff et al. [9] who found relatively increased vertical ground reaction force during the stance phase when the rider was sitting. This might suggest that IMU measurements can be used as a proxy for ground reaction forces during riding. A change in PDmax was seen with a lower maximum pelvic position reached after push off when the rider was concurrently rising (Fig 2B). Probably this occurs due to the downward momentum induced when the rider actively rises and counteracts the hindlimb push off. This mimics a push off lameness in that hindlimb and agrees with the increased downward force through the stirrups when rising in the saddle that has previously been described [17, 18]. HDmin was not significantly affected by rising trot on a straight line in agreement with Robartes et al. [10] (Fig 1A). HDmax showed increased maximum height reached by the head after push off of from the forelimb during which stance the rider was sitting (Fig 1B). This could be due to the rider interfering with the normal movement by raising the hand and thus encouraging the horse’s head in an upward direction through the bit as the rider rises.

### Effect of the circle

During lungeing, there was an increase in PDmin asymmetry, indicating less downward pelvic movement during the inner hindlimb stance, in agreement with Rhodin et al. [13] and Starke et al. [11] (Fig 2A). PDmax was not significantly affected by lungeing (Fig 2B). This is in accordance with a previous study where some horses reached a lower maximum pelvic height after outside (more common) and other horses after inside hindlimb stance [13]. In earlier studies, head parameters were less systematically affected by the circular track and similar to pelvic PDmax variably found to be attributed to the inner or outer forelimb in different horses [13]. A decreased downward movement of the head during inside forelimb stance has been shown in two studies [11–12] and decreased downward movement during outside forelimb stance was most common in another [13]. In the present study, there was no significant difference from ‘unridden straight’ for the two head variables during lungeing (Fig 1). The distribution around zero suggests that the head asymmetry parameters were variably attributed to the inner or outer forelimb in this study population, as is also seen in Rhodin et al. [13].

### Effect of the combination of circle and rising trot

The results of this study show that hindlimb movement asymmetry variables are influenced both by circular movement and rising trot. This supports our first hypothesis that rising trot and riding on a circle induce systematic changes in the movement symmetry of the horse. Rising on the correct diagonal on the circle (sitting during outside forelimb/inside hindlimb diagonal stance) induced a PDmin asymmetry of opposite direction to the circle induced asymmetry (Fig 2A). The horses in the present study became significantly more symmetric with the rider rising on the correct diagonal compared with lungeing in the same direction, which is in contrast to Robartes et al. [10]. Furthermore, rising on the incorrect diagonal on a circle induced the highest degree of asymmetry for all variables. Here we offer a biomechanical explanation which supports the traditional equestrian instruction that recommends the use of a certain ‘diagonal’ in rising trot on a circle. PDmax was distinctly more affected by the rising trot than by the circle (Fig 2B). In addition, similarly to the straight line, there was a decrease in pelvic height reached after the stance of the hindlimb during which the rider was sitting. Although unridden exercise on the circle did not induce changes for the head parameters (compared to in-hand straight line), there were some significant effects with the addition of a rider (Fig 1). HDmin for most of the ridden conditions showed similar findings to Starke et al. [11] with a reduced downward movement during the inside forelimb stance, mimicking an inner forelimb lameness. An explanation for this could be that the riders influence on the head by the reins or added weight equate the horses moving patterns on the circle from variably inner and outer forelimb attributed HDmin towards a more consistent inner forelimb HDmin.

### Changes in horses with pre-existing asymmetries

In general, the asymmetry measured during a given condition appeared to be a simple summation of the baseline asymmetry of the horse and the asymmetries induced by the rider and those induced by a circular track. This means that a horse with a PDmin asymmetry attributed to the right hindlimb will increase in amount of asymmetry on a straight line if the rider is seated during the left (sound) hindlimb stance, thus summating the baseline asymmetry with the asymmetry induced by the rising trot (Fig 4A). This contrasts with an orthopedic textbook where lameness is described to increase when sitting during lame hindlimb stance [19]; however, no reference is being made to the type of lameness or how the lameness had been evaluated or quantified. In the present study, the horses with a right hindlimb impact movement asymmetry (PDmin) had the highest degree of asymmetry when trotting on a right circle with the rider rising on the incorrect diagonal since the circle induced PDmin asymmetry [11, 13] is also added. This is in accordance with the summation between induced hindlimb lameness and circle induced asymmetry, as seen in Rhodin et al. [20]. A horse with a right hindlimb push off movement asymmetry (PDmax) will have an even further decreased upward pelvic movement (becoming more asymmetric) during rising trot when the rider is seated during the stance of the hindlimb with decreased push off (Fig 4B). Such horses show the highest degree of motion asymmetry when the limb with decreased push off is located to the outside of the circle and the rider rises on the incorrect diagonal. Both PDmin and PDmax also decrease during some conditions when the baseline asymmetries are counteracted by circle and/or rider induced asymmetries (Fig 4).

Both HDmin and HDmax asymmetries can be decreased by rising trot on one of the diagonals and by riding in a left or right circle (Fig 3). Accentuating a head asymmetry is harder. Horses with decreased downward head movement during the stance phase of one forelimb (HDmin asymmetry) significantly increase degree of asymmetry with that limb to the inside of a circle and the rider simultaneously rising on the incorrect diagonal. HDmax never increase significantly. In the present study, no significant increase for the head variables was seen during lungeing. This is in contrast to Pfau et al. [21], describing an increase with the lame forelimb to the inside of a circle, most prominently on the hard surface but also seen on a soft surface. On the contrary, Rhodin et al. [20] saw an increased asymmetry with the lame limb on the outside of the circle on a soft surface, although this finding was only significant for one of the directions. The difference in results between these studies and the present may be related to degree of asymmetry, which is small for these horses, or the nature of the asymmetry, which is unknown for the horses in the present study.

The demonstrated increase or decrease of pre-existing movement asymmetries depending on condition provides supporting evidence for the second hypothesis: rising trot on the incorrect diagonal on the circle will increase any HDmin/PDmin/PDmax asymmetries in the direction where the circle induced asymmetries exacerbate the initial asymmetry.

### Limitations of the study

The horses included in this study were all in full training, considered non-lame by their owner and had not been treated for lameness recently. It is unknown whether the initial asymmetries seen in the asymmetric subsets are caused by pain or could reflect biological variation. The degree of asymmetry exceeded the recommended thresholds for repeatable [15] and relevant asymmetries but were still low-grade. Additional data should be collected from horses with clinically diagnosed lameness in order to improve our understanding of the meaning and diagnostic potential of lungeing and riding exercises in the context of different underlying causes of lameness.

The same intermediate level rider was used to minimize possible variation between riders in order to study the influence of the different conditions on the vertical movement asymmetry of the horses. The use of only one rider means that caution has to be applied when aiming at generalizing the results, as riders can themselves be asymmetric in their movement patterns [22]. Such rider asymmetries may have contributed to some of the differences reported when comparing our findings to previously published data. Finally, all measurements were conducted on a soft surface. This represents a commonly used type of surface for training, competition [23] and ridden exercises during lameness work up but prevents generalization of results to other surface types.

## Conclusions

Rising trot, but not sitting trot or two point seat, induces systematic changes in vertical movement symmetry of head and pelvis in riding horses. The most prominent effect was decreased pelvic rise that probably occurs due to the downward momentum induced when the rider actively rises, counteracting the hindlimb push off. This mimics a push off lameness in the hindlimb of the diagonal the rider was sitting on in the rising trot. When ridden on a circle the asymmetry induced by rising trot on the correct diagonal counteracts circle induced asymmetry, rendering the horse more symmetrical. This offers an explanation to the equestrian principle of rising on the ‘correct diagonal’.

In horses with small pre-existing movement asymmetries, the asymmetry induced by rising trot and the circular track increases or decreases the horse’s baseline asymmetry depending on the sitting diagonal and direction on the circle. These findings suggests that trot on a circle and rising on the incorrect diagonal, highlights most pre-existing asymmetries on one of the directions. The knowledge of how to amplify pre-existing asymmetries in the horses may thus augment the sensitivity of the visual evaluation of low grade asymmetry. For hindlimb lameness, rising trot may be used to identify the type of lameness during subjective evaluation. A push off lameness (PDmax) would be expected to increase when the rider sits down during the lame hindlimb stance whereas an impact lameness (PDmin) would be expected to decrease. This needs to be investigated further in horses with a higher degree of asymmetry and diagnosed clinical lameness.

## Supporting information

S1 File Tables of renamed conditions for second mixed model. Excel sheet containing table of the renamed conditions for the second mixed model analysis (asymmetric subset).
(XLSX)

S2 File Output from the first mixed model. Excel sheet containing table of output from the first mixed model of all horses.
(XLSX)

S3 File Output from the second mixed model. Excel sheet containing table of output from the second mixed model using the subset of horses selected for asymmetry.
(XLSX)

S4 File Data sets used for the data analysis. Excel book containing sheets with the data sets used for the five different mixed model analyses preformed. Sheet ‘Abbreviations’ contains explanations of the variable names. Sheet ‘All_horses’, contains the data for the analysis including all horses. Sheet ‘HDmin_asymmetric’, ‘HDmax_asymmetric’, ‘PDmin_asymmetric’ and ‘PDmax_asymmetric’ contains the data from the asymmetric subsets selected for asymmetry of the respective symmetry variables.
(XLSX)
